# Multi-Steroid Profiling and Machine Learning Reveal Androgens as Candidate Biomarkers for Endometrial Cancer Diagnosis: A Case-Control Study

**DOI:** 10.3390/cancers17101679

**Published:** 2025-05-16

**Authors:** Marija Gjorgoska, Angela E. Taylor, Špela Smrkolj, Tea Lanišnik Rižner

**Affiliations:** 1 Institute of Biochemistry and Molecular Genetics, Faculty of Medicine, University of Ljubljana, 1000 Ljubljana, Slovenia; marija.gjorgoska@mf.uni-lj.si; 2 Institute of Metabolism and Systems Research, University of Birmingham, Birmingham B15 2TT, UK; a.taylor.5@bham.ac.uk; 3 Department of Gynecology and Obstetrics, Faculty of Medicine, University of Ljubljana, 1000 Ljubljana, Slovenia; spela.smrkolj@mf.uni-lj.si; 4 Division of Gynecology and Obstetrics, University Medical Centre, 1000 Ljubljana, Slovenia

**Keywords:** endometrial cancer, multi-steroid profiling, liquid chromatography–tandem mass spectrometry, machine learning, diagnosis, prognosis

## Abstract

Objective: To evaluate the diagnostic and prognostic potential of preoperative serum steroid levels in endometrial cancer (EC) alone and in combination with clinical parameters and biomarkers CA-125 and HE4. Methods: This single-center observational study included 62 patients with EC and 70 controls with benign uterine conditions who underwent surgery between June 2012 and February 2020. Preoperative serum levels of classic androgens, 11-oxyandrogens, glucocorticoids and mineralocorticoids were measured using liquid chromatography–tandem mass spectrometry (LC-MS/MS). Machine learning was used to assess their diagnostic and prognostic value alone and combined with clinical parameters and tumor biomarkers. Results: Patients with EC had significantly higher serum levels of classic androgens (androstenedione, testosterone), 11-oxyandrogens (11β-hydroxy-androstenedione, 11β-hydroxy-testosterone) and glucocorticoids (17α-hydroxy-progesterone, 11-deoxycortisol) compared to controls. While individual steroids had limited diagnostic value, a multivariate model including classic androgens, CA-125, HE4, BMI and parity achieved an AUC 0.87, 79.1% sensitivity and 74.7% specificity in distinguishing EC from benign uterine condition. This model outperformed our previously published model based on CA-125, HE4 and BMI (AUC: 0.81, *p* < 0.0001). Prognostically, HE4 was the strongest marker for lymphovascular space invasion (LVSI) (AUC: 0.79) and deep myometrial invasion (MI) (AUC: 0.71). Among steroids, androstenedione was the most predictive of LVSI (AUC: 0.67), while 11β-hydroxy-testosterone was the strongest predictor of deep MI (AUC: 0.64). Conclusions: Patients with EC exhibit distinct steroid hormone profiles. While steroids alone offer modest diagnostic and prognostic value, integrating them into multivariate models improves diagnostic accuracy.

## Simple Summary

Endometrial cancer (EC) is the second most common gynecologic malignancy, with its incidence rising due to demographic changes. Current diagnostic methods are invasive and insufficiently specific, highlighting the need for accurate, non-invasive biomarkers. In this study, we applied mass spectrometry-based multi-steroid profiling and machine learning to analyze systemic steroid levels—focusing on androgens, 11-oxyandrogens, glucocorticoids and mineralocorticoids—as potential diagnostic and prognostic biomarkers. Our cohort included 62 patients with EC and 70 controls with benign uterine conditions. We identified distinct steroid level alterations between cases and controls. While steroids alone had limited diagnostic and prognostic value, a multivariate model combining classic androgens, CA-125, HE4, BMI and parity achieved an AUC of 0.87, 79.1% sensitivity and 74.7% specificity in distinguishing EC from benign conditions. This model outperformed those based on CA-125, HE4 or their combination with BMI. These findings underscore the potential of integrating steroid profiling with established biomarkers to enhance EC detection.

## 1. Introduction

Endometrial cancer (EC) is the sixth most common cancer affecting women, with approximately 420,000 new cases diagnosed worldwide in 2022 [1]. Its incidence is rising globally, driven by factors such as population growth, aging, increasing obesity rates and a higher human development index [2]. Traditionally, EC was classified into two groups: type I (endometrioid) and type II (non-endometrioid, including serous, clear-cell and un/dedifferentiated EC) [3]. However, this classification does not align well with molecular characteristics, limiting its use in precision medicine. The Cancer Genome Atlas (TCGA) introduced over a decade ago a molecular classification that stratifies EC into four groups: POLE-altered (POLE-alt), mismatch repair deficient (dMMR), non-specific molecular profile (NSMP) and TP53-altered (TP53-alt) tumors [4]. The first three groups mostly include low-grade, clinically favorable endometrioid tumors, while TP53-alt tumors are typically high-grade endometrioid or serous with a higher risk of recurrence [4,5,6]. Both classification systems are currently used in clinical practice in Europe [5,7].

EC often presents early, typically with postmenopausal bleeding, but this red flag symptom is nonspecific, with only 5–10% of symptomatic women being ultimately diagnosed with the disease [8]. A definitive diagnosis requires histological examination of an endometrial tissue sample, usually obtained when transvaginal ultrasound shows an endometrial thickness greater than 5 mm. While this threshold is highly sensitive (over 90%), it has low specificity (around 50%) in postmenopausal women, leading to a high rate of false positives [9]. Therefore, there is a clear need for less invasive and more accurate diagnostic tools. The Risk of Endometrial Malignancy B (REM-B) algorithm, which combines age, presence of abnormal uterine bleeding, BMI, HE4 levels and ultrasound findings, shows excellent sensitivity and specificity (>90%) but requires further validation [10]. Promising diagnostic models using omics approaches (e.g., proteomics, metabolomics) and machine learning have also emerged [11,12,13,14]. However, no blood-based diagnostic tests for EC are currently included in European clinical guidelines [5].

Once EC is confirmed, preoperative biopsy findings, such as tumor type and grade guide surgical decisions [5]. Preoperative imaging, such as magnetic resonance imaging (MRI) aids in assessing myometrial and cervical involvement and detecting enlarged lymph nodes and disease spread [15]. However, risk stratification remains imprecise, as biopsy results often do not align with post-hysterectomy findings [16,17,18], highlighting the need for more accurate prognostic tools. A recent study found that molecular classification of preoperative biopsy samples, combined with imaging tests, predicts advanced disease more reliably than histotype or grade [19]. This molecular information can be used preoperatively to help tailor surgical treatment. Additionally, biomarkers such as cancer antigen 125 (CA-125) and human epididymis protein 4 (HE4) have been shown to improve preoperative risk stratification [20,21,22]. HE4 is also useful for predicting deep myometrial invasion (MI) [21,23] and lympho-vascular space invasion (LVSI) [23], though further validation in prospective studies is needed.

The present study had two main objectives: (1) to evaluate preoperative serum levels of steroids, including classic androgens, 11-oxyandrogens, glucocorticoids and mineralocorticoids, in patients with EC and control patients, and (2) to examine their diagnostic and prognostic potential as individual biomarkers and in combination with other steroids, CA-125, HE4 and patient clinical characteristics. For this purpose, we measured preoperative serum levels of steroid hormones in 62 patients with EC and 70 women with benign uterine conditions using liquid chromatography–tandem mass spectrometry (LC-MS/MS). Machine learning was applied to evaluate the diagnostic and prognostic utility of these hormones.

## 2. Materials and Methods

### 2.1. Study Population

This single-center case-control observational study included 132 women who underwent surgical treatment at the Department of Obstetrics and Gynecology, University Medical Centre Ljubljana, Slovenia, between June 2012 and February 2020. Based on clinical and histopathological findings, participants were stratified into two groups: patients with endometrial adenocarcinoma (*n* = 62) and a control group of women with benign uterine conditions, such as uterine prolapse or myomas (*n* = 70).

Inclusion criteria for the case group were age ≥40 years, a diagnosis of endometrial adenocarcinoma (any histotype, grade or International Federation of Gynecology and Obstetrics (FIGO) stage) and available serum sample for steroid quantification. For the control group, inclusion criteria were age ≥40 years, a diagnosis of benign uterine conditions and available serum sample for steroid quantification. Exclusion criteria for both groups included a diagnosis of polycystic ovary syndrome (PCOS), while the control group also excluded participants with endometrial hyperplasia. The study was approved by the Medical Ethics Committee of the Republic of Slovenia (Approval ID: 0120-487/2020/3) and all participants provided written informed consent prior to enrollment.

Patients were recruited by senior gynecologists with assistance from study nurses. Morning blood samples were collected 1 to 7 days before surgery, along with detailed information on lifestyle, medication use and gynecological and clinical status. Sample collection and processing followed a strict standard operating procedure specifically designed for metabolomic studies [24]. Blood samples (6 mL) were obtained via venipuncture using clot-activator tubes (BD Vacutainer, Franklin Lakes, NJ, USA, #368815). The samples were centrifuged at 2000 g for 15 min. Serum was carefully aspirated, divided into 200 µL aliquots and stored at −80 °C in 1.8 mL cryotubes (Nalgen Nunc International, Roskilde, Denmark, #375418) until analysis.

### 2.2. Multi-Steroid Profiling of Serum Samples by LC-MS/MS

Multi-steroid profiling was performed using an LC-MS/MS method developed and validated by Schiffer and collaborators at the Steroid Metabolome Analysis Core (SMAC) at the University of Birmingham [25].

#### 2.2.1. Sample Preparation

Steroids were extracted from serum samples by liquid–liquid extraction, as described previously [25,26]. Briefly, 180 µL of sample, calibrator or quality control (QC) was mixed with 10 µL of the internal standard mixture, followed by protein precipitation with 50 µL of acetonitrile (#75-05-8, Biosolve, Dieuze, France). Steroid extraction was performed with 1 mL tert-butyl methyl ether (MTBE) (#1634-04-4, Acros Organics, Fisher Scientific UK Ltd., Loughborough, UK), the mixtures were vortex-mixed at 1000 rpm for 10 min and the phases were allowed to separate for a further 20 min. Subsequently, the organic phase was transferred to a 96-well plate (Porvair Sciences Ltd., Wrexham, UK) with 700 µL glass inserts (Randox, Crumlin, UK) and dried under a nitrogen stream at 45 °C. The dried extract was reconstituted in 125 µL of 50% methanol (#136841, Biosolve, Dieuze, France) and water (#232141, Biosolve, Dieuze, France) prior to LC-MS/MS analysis.

#### 2.2.2. LC-MS/MS Analysis

LC-MS/MS analysis was performed on a Waters Acquity UHPLC (Waters Ltd., Wilmslow, UK) coupled to an XEVO TQ XS mass spectrometer (Waters Ltd., Wilmslow, UK). The detailed method description can be found in the original article [25]. Briefly, chromatographic separation was performed using a Phenomenex Luna Omega column, 1.6 µm, polar C18, 100 Å, 2.1 × 50 mm (Phenomenex, Macclesfield, UK) at 60 °C. Ten microliters of the reconstituted sample was injected. A linear gradient from 45% to 75% of mobile phase B (UHPLC grade methanol) was applied for five minutes at a flow rate of 0.6 mL/min, followed by a washing step with 95% B for 5.2 min and equilibration at starting conditions until injection of the next sample. Ammonium fluoride (6 mM) was introduced into the flow path via post-column infusion at a rate of 5 µL/min to enhance ionization. The autosampler was maintained at 10 °C. The eluate was then injected into the mass spectrometer operated in positive electrospray ionization (ESI) mode. The ion source temperature was 150 °C, the desolvation temperature and gas flow were 600 °C and 1200 L/h, respectively, and the cone gas was 150 L/h.

16 steroids were quantified, including progesterone (Chemical Abstracts Service (CAS) number: CAS: 57-83-0; #P8783, Sigma Aldrich GmbH, Roedermark, Germany), dehydroepiandrosterone (DHEA; CAS: 53-43-0; #D4000, Sigma Aldrich GmbH, Roedermark, Germany), androstenedione (A4, CAS: 63-05-8; #46033, Sigma Aldrich GmbH, Roedermark, Germany), testosterone, (T; CAS: 58-22-0; #T6147, Sigma Aldrich GmbH, Roedermark, Germany), 5α-dihydrotestosterone (DHT; CAS: 521-18-6; #A8380, Sigma Aldrich GmbH, Roedermark, Germany), 11β-hydroxy-androstenedione (11OHA4, CAS: 382-44-5; #A3009, Sigma Aldrich GmbH, Roedermark, Germany), 11-keto-androstenedione (11KA4, CAS: 382-45-6; #284998, Sigma Aldrich GmbH, Roedermark, Germany), 11β-hydroxy-testosterone (11OHT; CAS: 1816-85-9; #A5760, Sigma Aldrich GmbH, Roedermark, Germany), 11-keto-testosterone (11KT, CAS: 564-35-2; #A6720, Steraloids, Newport, R.I., USA), 17α-hydroxy-progesterone (CAS: 68-96-2; #H5752, Sigma Aldrich GmbH, Roedermark, Germany), 11-deoxycortisol (CAS: 152-58-9; #R0500, Sigma Aldrich GmbH, Roedermark, Germany), cortisol (CAS: 50-23-7; #H4001, Sigma Aldrich GmbH, Roedermark, Germany), cortisone (CAS: 53-06-5; #C2755, Sigma Aldrich GmbH, Roedermark, Germany), 11-deoxycorticosterone (CAS: 64-85-7; #D6875, Sigma Aldrich GmbH, Roedermark, Germany), corticosterone (CAS: 50-22-6; #C2505, Sigma Aldrich GmbH, Roedermark, Germany) and aldosterone (CAS: 52-39-1; #Q2000-000, Steraloids, Newport, R.I., USA).

Data processing and quantification were performed using TargetLynx software version 4.2 (Waters Ltd., Wilmslow, UK). For T, 3% of the samples were below the lower limit of quantification (LLOQ), for 11-deoxycortisol, 3.8%, for 11OHT, 4.6%, and for 17α-hydroxy-progesterone, 12.12%. In these cases, values below the LLOQ were assigned as LLOQ/2. The analytes 11-deoxycorticosterone, DHT, aldosterone and progesterone were below the LLOQ in 95%, 83%, 79% and 74% of samples, respectively, and were not analyzed further.

### 2.3. Measurement of Serum CA-125 and HE4 Levels

Serum levels of CA-125 and HE4 for samples collected from June 2012 to December 2014 were previously determined by our group and are reported by Knific and collaborators [23]. Specimens collected from December 2014 to February 2020 were analyzed separately for the present study. In both cases, analysis of CA-125 and HE4 levels was performed at the Clinical Institute for Clinical Biochemistry, University Medical Center, Ljubljana, using clinically validated electroluminescent immunoassays (ECLIAs) specific for CA-125 (REF: 11776223190, Roche Diagnostics GmbH, Mannheim, Germany) and HE4 (REF: 05950929190, Roche Diagnostics GmbH, Mannheim, Germany), on a Cobas e411 analyzer (Roche Diagnostics GmbH, Mannheim, Germany).

### 2.4. Statistical Analysis

The data were numerically anonymized and were collected in Microsoft Office Excel 2010 spreadsheets. Statistical analysis was performed in R studio version 4.3.0 or higher. Probability of <0.05 was defined as statistically significant and all tests were two sided. Continuous variables were expressed as median values with interquartile range (IQR) and were compared using the Mann–Whitney U test. Categorical variables were expressed as frequencies with percentages and compared using the Chi-square or the Fisher exact test. Power analysis was performed to assess the adequacy of the sample size for group comparisons using GPower version 3.1. The power for detecting small, medium and large effect sizes (Cohen’s d = 0.2, 0.5, 0.8) between cases and controls was calculated as 20%, 79.4% and 99.4%, respectively, indicating sufficient power to detect medium to large effects.

Machine learning was performed in R Studio (version 4.3.0 or higher) using caret library [27]. We tested 21 variables, including 12 steroid hormones (DHEA, A4, T, 11OHA4, 11OHT, 11KA4, 11KT, 17α-hydroxy-progesterone, 11-deoxycortisol, cortisol, cortisone and corticosterone); 3 steroid combinations, including classic androgen pool (sum of DHEA, A4 and T), 11-oxyandrogen pool (sum of 11OHA4, 11OHT, 11KA4 and 11KT) and glucocorticoid pool (sum of 17α-hydroxy-progesterone, 11-deoxycortisol, cortisol and cortisone); 2 proteins (HE4 and CA-125); and 4 clinical characteristics (age, menopause status, BMI and parity).

All continuous variables were first ln-transformed for normality, followed by standardization (mean subtraction and division by standard deviation) to ensure comparability across different variables. Logistic regression models, both univariate and multivariate, were trained using a 5 × 5-fold cross-validation scheme, with 1000 iterations to ensure stability and robustness. For the multivariate logistic regression models, first we employed feature selection using the function stepAIC() from MASS library in both directions to identify the most informative variables associated with the classification outcome.

The minimum sample size for the logistic regression models was calculated based on Peduzzi et al. [28] using the formula: N = 10 × k/p, where N is the minimum required number of samples, k is the number of covariates and p is the smallest proportion of negative or positive cases in the cohort. Using this approach, we calculated the minimum sample size needed for the diagnostic univariate logistic regression models to be 22, for the most complex multivariate diagnostic model (five-variable logistic regression model) to be 106 samples. Our cohort of 132 patients exceeded this minimum, ensuring adequate power for the diagnostic models.

In addition to cross-validation, internal validation was performed via bootstrapping to assess the robustness of the models. We generated 1000 bootstrap samples with replacement from the original dataset and refitted the logistic regression models on each resample to estimate the variability in model performance.

Subset analysis was performed to distinguish between patients without and with LVSI, without and with deep MI, and between low- and high-grade tumors, using the same variables as for the diagnostic models. The prognostic models for LVSI were evaluated using 5 × 5 cross-validation on a dataset of 12 positive and 50 negative cases. The prognostic models for deep MI were tested using 5 × 5 cross-validation on 16 positive and 44 negative cases (data were unavailable for 2 patients). The prognostic models for tumor grade prediction were evaluated on 46 low-grade cases and 16 high-grade cases using 5 × 5 cross-validation. Due to small sample sizes and class imbalance, only univariate logistic regression was applied. For the univariate prognostic models, the calculated minimum sample sizes were 52 patients for LVSI status and 37 for deep MI status. In both cases, the number of samples in the sub-cohort exceeded the calculated minimum, ensuring sufficient power for these models as well.

We assessed model performance using AUC (area under the receiver operating (ROC) curve), sensitivity, specificity, precision, F1 score, accuracy and Akaike information criteria (AIC). ROC curves were used for visualization of classification performance.

## 3. Results

### 3.1. Description of the Cohort

**Figure 1:**
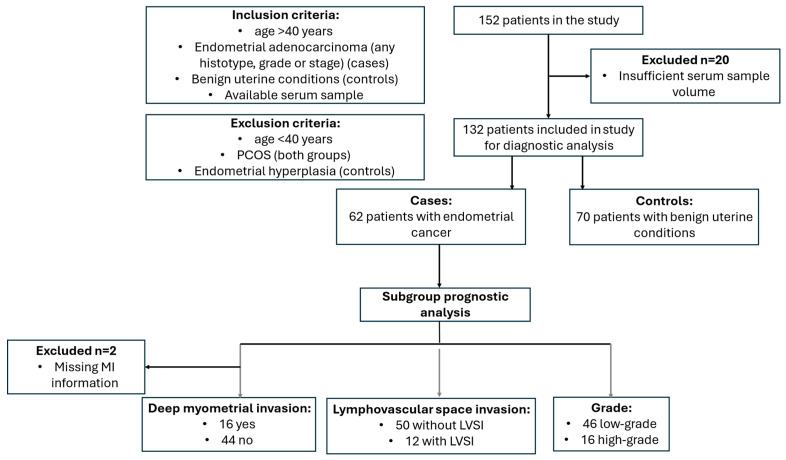
Study flow diagram. LVSI, lymphovascular space invasion; MI, myometrial invasion.

In this study, we included 62 patients with endometrial cancer (EC) and 70 women with benign uterine conditions (Figure 1). The median age at diagnosis was 64.5 years (interquartile range [IQR]: 59.3–71.0) in the case group and 64.0 years (IQR: 55.0–71.0) in the control group. The median body mass index (BMI) was 30.4 kg/m^2^ (IQR: 27.2–35.2) for the EC group and 27.4 kg/m^2^ (range: 23.8–30.5) for the control group (*p* = 0.005). No significant differences were found between the groups in terms of age, menopausal status, presence of type 2 diabetes, arterial hypertension, use of hormonal therapy, medication intake or smoking status (Table 1). In terms of tumor biomarkers, the median CA-125 level was 13.0 kU/L (IQR: 9.1–19.0 kU/L) in the control group, compared to 21.7 kU/L (IQR: 13.4–34.5 kU/L) in the case group (*p* < 0.0001). The median HE4 level in the control group was 55.6 pmol/L (IQR: 45.6–69.7 pmol/L), while in the case group it was 86.0 pmol/L (IQR: 64.7–130.4 pmol/L) (*p* < 0.0001). None of the patients received neoadjuvant chemotherapy.

Among the patients with EC, 55 had endometrioid EC, 6 had serous EC and 1 had clear-cell EC. The average number of lymph nodes removed during lymphadenectomy was 19.36 (range: 1–41), with an average of 4.4 tumor-positive lymph nodes (range: 1–13, *n* = 5). Fifty-four patients had no tumor-positive lymph nodes, and for three patients, this information was missing. LVSI was identified in 12 patients (19%). MI was classified as follows: deep invasion (more than 50% of the myometrium) in 16 patients (26%), less than 50% invasion in 30 patients (48%) and no invasion in 14 patients (23%). Information on MI was missing for 2 patients. Based on the FIGO classification, the distribution of EC stages was as follows: Stage IA (*n* = 40, 64.5%), Stage IB (*n* = 11, 17.7%), Stage IIA (*n* = 1, 1.6%), Stage IIB (*n* = 1, 1.6%), Stage IIIA (*n* = 1, 1.6%), Stage IIIB (*n* = 1, 1.6%), Stage IIIC (*n* = 2, 3.2%) and Stage IVB (*n* = 3, 4.8%). Data on FIGO stage were missing for two patients. Detailed histopathological characteristics are provided in Supplementary Table S1.

### 3.2. Preoperative Steroid Hormone Levels Differ Between Patients with EC and Women with Benign Uterine Conditions

We used a validated LC-MS/MS method to determine preoperative serum levels of steroid hormones, including classic and 11-oxyandrogens, glucocorticoids, mineralocorticoids and progesterone (see Supplementary Figure S1 for steroid synthesis pathways).

Univariate statistical analysis identified significant differences in six steroid hormones between patients with EC and controls (Table 2). More specifically, levels of 11β-hydroxylated androgens (11OHA4, 11OHT) and their classical androgen counterparts (A4, T, respectively) were significantly higher in cases compared to controls (*p* = 0.01–0.03). Likewise, glucocorticoid precursors 17α-hydroxy-progesterone and 11-deoxycortisol were also elevated in cases compared to controls (*p* = 0.04 and 0.01, respectively), while cortisol levels showed a borderline increase (*p* = 0.055).

### 3.3. Preoperative 11-Oxyandrogen Levels Differ Between Tumor Grades

We next investigated the association between steroid hormone levels and key clinicopathological features of EC, including histological subtype, tumor grade, metastasis, LVSI and deep MI.

Regarding histological subtypes, we observed significant differences in 11KA4 (*p* = 0.014) and CA-125 (*p* = 0.006) levels between endometrioid and serous EC. Additionally, 11OHA4 levels were higher in serous cases, though this difference was borderline significant (*p* = 0.059) (Supplementary Table S2).

When analyzing tumor grade, we found that high-grade tumors (grade 3 endometrioid and non-endometrioid EC) exhibited significantly higher levels of 11OHA4 (*p* = 0.012), 11KA4 (*p* = 0.008), CA-125 (*p* < 0.001) and HE4 (*p* = 0.005) compared to low-grade tumors (grade 1–2 endometrioid EC). Within the endometrioid subgroup, steroid hormone levels did not differ significantly between low- and high-grade tumors, although CA-125 (*p* = 0.003) and HE4 (*p* = 0.013) remained elevated in high-grade cases (Supplementary Table S2).

In terms of metastasis, LVSI and deep MI, we found no significant differences in steroid hormone levels between affected and unaffected patients (Table 3). However, CA-125 and HE4 were significantly elevated in metastatic cases (*p* = 0.002 and *p* = 0.001, respectively) and in patients with LVSI (*p* = 0.018 and *p* = 0.001, respectively). Additionally, HE4 levels were significantly higher in cases with deep MI (*p* = 0.004).

### 3.4. Development of Machine Learning Diagnostic Models Based on Preoperative Serum Steroid Levels

We applied machine learning to evaluate the diagnostic and prognostic potential of steroid hormones, steroid pools (classic androgens, 11-oxyandrogens, glucocorticoids) and their combinations with CA-125, HE4 and clinical parameters (age, BMI, menopause status, parity). The models’ performance was evaluated with cross-validation.

**Figure 2:**
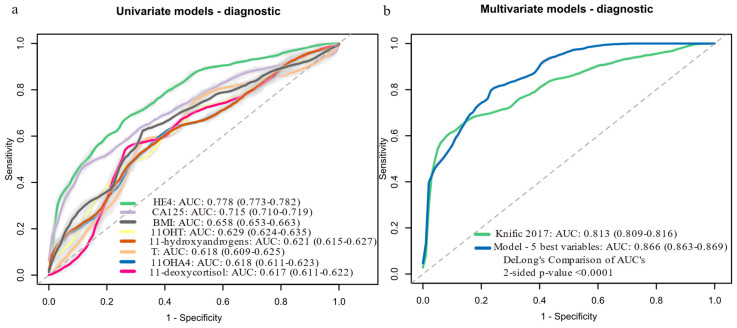
Logistic regression models for predicting EC probability (cases, *n* = 62; controls, *n* = 70): (**a**) Univariate models with AUC above 0.6. (**b**) Multivariate models: Knific 2017 [23] = 0.884 (ln[CA-125]) + 2.402 (ln[HE4]) + 3.142 (ln[BMI]) − 23.284; model with 5 best variables = 2.868 (ln[HE4]) + 1.116 (ln[Androgen pool]) + 2.929 (ln[BMI]) − 0.835 (Parity) + 1.292 (ln[CA-125]) − 27.214. Data in brackets represent 95% confidence intervals (CI). Light grey color around the ROC curves shows standard deviation. 11OHA4, 11β-hydroxy-androstenedione; 11OHT, 11β-hydroxy-testosterone; AUC, area under the receiver operating curve; BMI, body mass index; CA-125, cancer antigen 125; HE4, human epididymis protein 4; ln, natural logarithm; ROC, receiver operator curve; T, testosterone.

In univariate analysis, HE4 was the strongest EC predictor (AUC: 0.778, 95% CI: 0.773–0.782), while among steroids, 11OHT performed best (AUC: 0.629) (Figure 2a, Supplementary Table S3). The performance of other steroids is given in Supplementary Table S3. For multivariate analysis, we first used a stepwise approach to identify the top predictive variables. The best two-variable model (HE4 + classic androgen pool) performed similarly to the Knific et al. model based on CA-125, HE4 and BMI (AUC: 0.811 vs. 0.813, *p* = 0.881). Adding BMI, parity and CA-125 to the best two-variable model sequentially improved performance, with the final five-variable model (HE4, classic androgen pool, BMI, parity and CA-125) achieving the highest AUC (0.866, 95% CI: 0.863–0.869), sensitivity (79.1%) and specificity (74.7%), significantly outperforming CA-125 alone (*p* < 0.0001), HE4 alone (*p* = 0.004) and the Knific et al. model (*p* < 0.0001) (Figure 2b). The Knific et al. model’s performance in our cohort (AUC: 0.813) differed slightly from the original study (AUC: 0.804), likely due to differences in 20 samples between this cohort and the original one.

To further validate model robustness, we performed internal validation using bootstrapping (1000 resamples) (Supplementary Table S4). The five-variable model performance remained robust (mean AUC: 0.872; 95% CI: 0.809–0.924; mean sensitivity: 79.4%; mean specificity: 75.5%). The Knific et al. model yielded a bootstrap AUC of 0.822 (95% CI: 0.747–0.891). These results support the stability of our findings across different validation approaches.

### 3.5. Development of Machine Learning Prognostic Models Based on Preoperative Serum Steroid Levels

**Figure 3:**
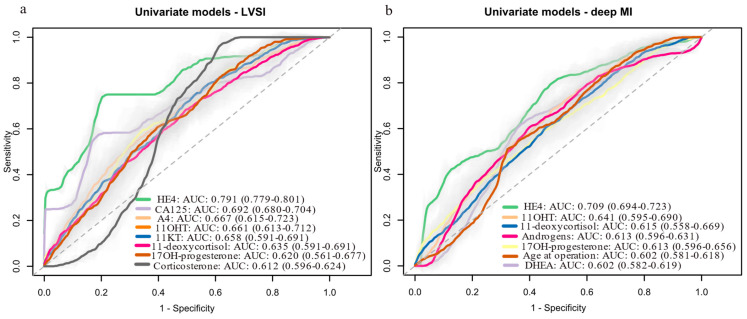
Univariate logistic regression models for predicting: (**a**) LVSI status (LVSI-positive: *n* = 12; LVSI-negative: *n* = 50), (**b**) deep MI (deep MI present: *n* = 16; absent: *n* = 44). Data are shown for variables with AUCs above 0.6. Data in brackets represent 95% confidence intervals (CI). Light grey color around the ROC curves shows standard deviation. 11OHT, 11β-hydroxy-testosterone; 11KT, 11-ketotestosterone; AUC, area under the receiver operating curve; CA-125, cancer antigen 125; DHEA, dehydroepiandrosterone; HE4, human epididymis protein 4; LVSI, lympho-vascular space invasion; MI, myometrial invasion; ROC, receiver operator curve; T, testosterone.

We also evaluated the prognostic potential of steroid hormones for predicting LVSI, deep MI and tumor grade. The models’ performance was evaluated with cross-validation. Steroid hormones alone had modest predictive value for LVSI (Figure 3a, Supplementary Table S5), deep MI (Figure 3b, Supplementary Table S6) and tumor grade (Supplementary Table S7) (AUC < 0.70). HE4 was the strongest predictor of LVSI (AUC: 0.791) and deep MI (AUC: 0.709), while A4 (AUC: 0.667) and 11OHT (AUC: 0.641) were the top-performing steroids, respectively. Differences in HE4′s performance for LVSI (AUC: 0.810) and deep MI (AUC: 0.776) compared to the Knific et al. study [23] were most likely due to differences in 20 samples between this cohort and the original one. For tumor grade, CA-125 was the best predictor (AUC: 0.810), with 11KA4 as the top-performing steroid (AUC: 0.690).

## 4. Discussion

In this study, we profiled preoperative serum steroid levels in 62 patients with EC and 70 women with benign uterine pathologies using LC-MS/MS. To our knowledge, this is the first study to evaluate the diagnostic and prognostic potential of systemic steroids in EC using machine learning. This is also the first case-control study evaluating systemic 11-oxyandrogen levels in EC.

We found that patients with EC exhibit distinct alterations in systemic steroid hormone profiles, including elevated levels of classic androgens, 11-oxyandrogens and glucocorticoids. In particular, the marked increase in 11β-hydroxylated androgens could be the result of enhanced CYP11B1 activity, an adrenal-specific enzyme that catalyzes the 11β-hydroxylation of A4 and T and is also responsible for cortisol synthesis. Indeed, we observed concurrent elevations of 11OHA4, 11OHT, cortisol and corticosterone (a CYP11B1/2 product) in cases supporting the hypothesis of a systemic adrenal response to tumor-related stress or immune activation.

An additional explanation may lie in the role of gut microbiota, which is increasingly recognized for its involvement in steroid metabolism. Microbial enzymes can generate androgens such as DHEA and T from precursors like pregnenolone and 17α-hydroxy-pregnenolone [29] and may also contribute to the production of 11-oxyandrogens from cortisol or other C21 steroids [30,31,32]. The distinct alterations in the gut microbiota in patients with EC [33], might contribute to altered systemic steroid levels as well, although this need to be investigated further.

A third explanation of altered 11-oxyandrogen levels could be the tumor itself. Although Dahmani et al. reported that systemic levels of 11-oxyandrogens decline after tumor removal, suggesting tumor-driven regulation [34], our recent findings show that CYP11B1 is not expressed in EC tumors or in vitro cell lines [35]. We also showed that local intra-tumoral production of 11-oxyanrogens cannot happen from classic androgen precursors [35]. Consequently, the postoperative drop in systemic 11OHA4 and 11OHT levels cannot be solely attributed to tumor excision and likely reflects larger systemic changes.

Finally, the adipose tissue can be an important regulator of steroid hormone levels. Given that higher BMI is characteristic of patients with EC [36], it is plausible that expanded adipose depots in these individuals further drive alterations in both classic and 11-oxyandrogen levels. Indeed, the adipose tissue has been shown to express steroid metabolizing enzymes, like AKR1C3 [37,38,39].

Moving on to steroid diagnostic potential, we found that individual serum steroids had limited diagnostic accuracy, but adding them to CA-125, HE4 and BMI significantly improved both sensitivity and specificity. This biologically meaningful enhancement suggests that including steroid hormones provides a more complete biochemical signature than CA-125 and HE4, as steroids offer a direct readout of the endocrine dysregulation and intracrine activity that underpin EC pathogenesis. These diagnostic improvements align with extensive epidemiological data showing androgens, like DHEA-sulfate [40,41], A4 [40,41], T [41] and some androgen glucuronides [40] to be elevated in patients with EC, although not all studies agree [40,42,43]. Moreover, endometrial tumors, especially endometrioid ones, are known to express steroid-metabolizing enzymes and receptors [44,45,46,47,48,49,50], and to form locally androgens, 11-oxyandrogens and estrogens [35,51], suggesting that this intra-tumoral activity may also contribute to the improved diagnostic performance of the models.

Regarding prognostic potential, we found that individual serum steroids had limited prognostic accuracy for LVSI and deep MI. Unfortunately, due to the limited sample size of this sub-cohort, we were unable to investigate them in a multi-variate manner. The best prognostic predictors of LVSI and MI in our cohort were HE4 and CA-125, consistent with prior studies [21,22,52,53,54,55,56] and systematic reviews and meta analyses [57,58], which report strong performance of these markers for LVSI and MI prediction.

Several lines of evidence provide a rationale for why steroid hormones should be taken into consideration into prognostic models. For example, Tangen et al. reported that higher preoperative plasma levels of DHEA, DHEA-sulfate, progesterone (and its 21-hydroxylated metabolite) and estrone sulfate were linked to longer overall survival in patients with EC [59]. Conversely, lower concentrations of 17-OH-progesterone, 11-deoxycortisol and A4 correlated with more aggressive tumor features and poorer disease-specific survival [60]. Moreover, 11-oxyandrogen metabolites appear to carry independent prognostic information: elevated preoperative 11-ketoandrosterone predicted a higher risk of recurrence, while increased postoperative 11-hydroxyandrosterone was associated with both greater recurrence rates and reduced survival [34].

Beyond their diagnostic and prognostic potential, changes in the systemic steroid profile of patients with EC also have significant implications for tumor biology. Forsse and colleagues found that tumors from patients with low plasma levels of 17α-hydroxyprogesterone and 11-deoxycortisol had increased expression of genes related to cell proliferation, while tumors from patients with high plasma levels of these hormones showed upregulated estrogen signaling and increased expression of genes associated with inflammation [60]. Additionally, estradiol levels were positively correlated with the expression of genes involved in estrogen receptor signaling within tumors [59,60]. Our recent work also highlighted differences in the intra-tumoral metabolism of 11-oxyandrogen precursors (11OHA4 and 11KA4) between low- and high-grade EC tumors. Low-grade tumors were more efficient in producing 11KT, a metabolite capable of activating the androgen receptor, which has been associated with better survival outcomes [35].

Currently, there are a considerable number of studies focusing on biomarkers for EC detection and prognosis, although most of these studies are in discovery phase, and only a few have been validated in independent, multi-centric cohorts. These studies explore a wide range of biomolecules across various biological fluids—including plasma, serum, cervicovaginal fluid and uterine aspirates—and increasingly apply machine learning approaches (reviewed in [61,62]). Among the biomolecules studied are proteins (hormones, cancer-associated antigens, plasma glycoproteins, plasma lipoproteins, enzymes, growth factors) [11,63,64,65,66,67,68,69], lipids and metabolites (glycerophospholipids, sphingolipids, fatty acids, amino acids) ([12,14,70,71,72,73,74,75,76,77,78,79,80,81]), micro RNAs [82,83], circulating tumor DNA [84,85,86]. Additionally, routine blood count parameters—reflecting systemic immune or inflammatory responses—have shown promising diagnostic potential [87,88,89].

Among these studies, two approaches currently show the greatest potential for clinical implementation: the REM-B algorithm [10] and the metabolomic panel developed by Troisi et al. [81]. The REM-B algorithm integrates patient age, presence of abnormal uterine bleeding, BMI, HE4 levels and ultrasound findings and achieves a sensitivity and specificity of over 90% It has already been validated in an independent cohort, but multicenter validation is still needed to confirm its clinical utility. Similarly, the ensemble models based on metabolomic panel by Troisi et al. show a remarkable diagnostic performance with a sensitivity of 100% and specificity of 96% in detecting EC.

In our study we identified androgens as novel biomarker candidates for EC diagnosis. When incorporated into more comprehensive diagnostic models, these biomarkers can help improve diagnostic accuracy. Nonetheless, our study has several limitations. First, although the selection criteria for this study were rigorous, some potential biases may have influenced the results. More specifically, all study participants were from a single clinical center and primarily of Slovenian ethnicity, which may limit the generalizability of the findings to other populations with greater ethnic and demographic diversity. Additionally, the under-representation of cases with high-grade EC could introduce bias, as this may not fully reflect the disease spectrum seen in broader clinical settings. Second, measurement bias should be taken into consideration: although all samples were collected in the morning to minimize circadian effects, residual time-of-day variation may persist. To address this, we adhered to standardized protocols for sample collection, randomized sample processing and blinded analysts to clinical data ensuring that the analysis remained objective and free from potential interpretive bias. Furthermore, steroid measurements were based on a single preoperative time point, and finally, LC-MS/MS technical constraints limited the quantification of low-abundance hormones like DHT and progesterone.

Despite these limitations, our study also has notable strengths, such as including patients with benign uterine conditions as a control group, rather than healthy women, which are unlikely to require differential diagnosis. Moreover, we employed a multiplexed mass spectrometry method for steroid quantification, which is currently the gold standard for steroid analysis. Finally, the integration of steroids and proteins into diagnostic models represents a novel approach that could be easily translated into clinical practice once validated in larger, multi-center cohorts.

## 5. Conclusions

Our study shows that patients with EC have distinct steroid hormone profiles compared to women with benign uterine conditions. While individual steroid hormones had limited diagnostic value, combining them in multivariate models significantly improved diagnostic performance. Our multivariate model, incorporating classic androgens, CA-125, HE4, BMI and parity, achieved AUC value of 0.87, 79.1% sensitivity and 74.7% specificity in distinguishing EC from benign uterine conditions, significantly outperforming CA-125 and HE4 alone, or their combination with BMI. This highlights the potential of steroid profiling as a valuable addition to EC diagnostics.

Integrating steroid profiling into routine diagnostics could enhance early detection and improve patient outcomes; however, further validation in larger independent cohorts is needed to confirm the clinical utility of the proposed diagnostic model.

## Figures and Tables

**Table 1:** Clinical characteristics of the study participants.

Variable (Median (IQR) or *n*(%))	Controls (*n* = 70; 100%)	Cases (*n* = 62; 100%)	*p*-Value ^‡^
**Age (years)**	64.0 (55.0–71.0)	64.5 (59.3–71.0)	0.624
BMI (kg/m^2^)	27.4 (23.8–30.5)	30.4 (27.2–35.2)	**0.005**
**BMI category**			
Normal (<25 kg/m^2^)	25 (35.7%)	14 (22.6%)	0.055
Overweight (25–30 kg/m^2^)	26 (37.2%)	19 (30.6%)	
Obese (≥30 kg/m^2^)	19 (27.1%)	29 (46.8%)	
**Menopausal status**			
Premenopausal	8 (11.4%)	2 (3.2%)	0.148
Postmenopausal	62 (88.6%)	60 (96.8%)	
**Diabetes type 2**			
Yes	10 (14.3%)	9 (14.5%)	0.900
No	60 (85.7%)	52 (83.8%)	
Missing data	0 (0%)	1 (1.6%)	
**Arterial hypertension**			
Yes	40 (57.1%)	38 (61.3%)	0.473
No	30 (42.9)	23 (37.1%)	
Missing data	0 (0%)	1 1.6%)	
**Hormonal therapy in the past**			
Yes	2 (2.9%)	1 (1.6%)	0.100
No	67 (95.7%)	59 (95.2%)	
Missing data	1 (1.4%)	2 (3.2%)	
**Medication intake**			
Yes	46 (65.7%)	45 (72.6%)	0.508
No	24 (34.3%)	17 (27.4%)	
**Smoking status**			
Nonsmoker	45 (64.3%)	45 (72.6%)	0.170
Ever-smoker	20 (28.6%)	11 (17.7%)	
Missing data	5 (7.1%)	6 (9.7%)	
**Tumor biomarkers**			
CA-125 (kU/L)	13.0 (9.1–19.0)	21.7 (13.4–34.5)	**<0.001**
HE4 (pmol/L)	55.6 (45.6–69.7)	86.0 (64.7–130.4)	**<0.001**

^‡^ *p*-values for continuous variables were calculated with an independent samples median test; *p*-values for categorical variables were calculated with a Chi-square test or Fisher’s exact test. *p* values below the significance threshold 0.05 are bolded. Categories of body mass index (BMI) according to World Health Organization (WHO) Guidelines. CA-125, Cancer Antigen 125; HE4, human epididymis protein 4; IQR, interquartile range.

**Table 2:** Preoperative serum concentrations of the measured steroid hormones in patients with EC and control women with benign uterine pathologies.

	Controls (*n* = 70; 100%)		Cases (*n* = 62; 100%)		*p*-Value
Analyte	Median	IQR	Median	IQR	
**11-oxyandrogens (nM)**					
11OHA4	13.29	10.30–17.56	16.62	11.88–21.14	**0.010**
11KA4	5.84	4.31–6.99	5.40	3.78–7.95	0.810
11OHT	0.75	0.49–1.06	1.00	0.66–1.48	**0.006**
11KT	1.64	1.11–2.02	1.73	1.10–2.20	0.564
**Classic androgens (nM)**					
DHEA	9.29	6.96–14.39	10.98	7.53–18.14	0.123
A4	2.99	2.28–4.06	3.67	2.70–4.68	**0.029**
T	0.77	0.58–1.30	1.18	0.81–1.65	**0.010**
**Glucocorticoids (nM)**					
17α-hydroxy-progesterone	0.95	0.66–1.42	1.28	0.83–1.85	**0.041**
11-deoxycortisol	1.07	0.73–1.60	1.66	0.93–2.37	**0.012**
Cortisol	370.3	257.70–495.30	420.7	298.60–602.10	0.055
Cortisone	64.62	53.27–75.86	64.98	55.73–72.94	0.995
**Mineralocorticoids (nM)**					
Corticosterone	9.45	5.69–16.96	11.54	6.16–22.54	0.127

*p*-values were calculated using the non-parametric Mann–Whitney U test. *p* values below the significance threshold 0.05 are bolded. 11OHA4, 11β-hydroxy-androstenedione; 11OHT, 11β-hydroxy-testosterone; 11KA4, 11-keto-androstenedione; 11KT, 11-keto-testosterone; A4, androstenedione; DHEA, dehydroepiandrosterone; IQR, interquartile range; T, testosterone.

**Table 3:** Clinicopathological characteristics related to median steroid hormone levels (interquartile range) in patients with EC. Statistically significant *p*-values (<0.05) in bold.

	LVSI			Deep MI ^a^		
Analyte (Median (IQR))	Negative (*n* = 50; 81%)	Positive (*n* = 12; 19%)	*p*-Value	No (*n* = 44, 73%)	Yes (*n* = 16, 27%)	*p*-Value
**11-oxyandrogens (nM)**						
11OHA4	16.40 (11.75–20.61)	19.51 (14.84–29.40)	0.187	16.77 (10.80–21.71)	16.89 (15.26–21.88)	0.483
11KA4	5.07 (3.76–7.82)	5.95 (5.19–8.33)	0.269	4.85 (3.72–7.98)	6.04 (4.72–8.05)	0.367
11OHT	0.99 (0.65–1.42)	1.18 (0.74–1.48)	0.782	1.00 (0.63–1.53)	0.97 (0.76–1.44)	0.848
11KT	1.67 (1.10–2.20)	1.88 (1.10–2.15)	0.838	1.78 (0.92–2.31)	1.73 (1.23–1.97)	0.821
**Classic androgens (nM)**						
DHEA	11.25 (7.72–18.17)	9.82 (5.98–15.31)	0.364	11.73 (7.91–20.01)	9.16 (7.25–12.27)	0.116
A4	3.61 (2.79–4.68)	4.31 (2.49–4.68)	0.972	3.67 (2.79–5.12)	3.53 (2.64–4.40)	0.559
T	1.19 (0.84–1.65)	1.05 (0.65–1.28)	0.402	1.15 (0.79–1.65)	1.14 (0.77–1.27)	0.353
**Glucocorticoids (nM)**						
17α-hydroxy-progesterone	1.28 (0.82–1.72)	1.24 (0.87–1.99)	0.762	1.25 (0.82–1.88)	1.41 (0.84–1.80)	0.763
11-deoxycortisol	1.64 (0.83–2.37)	1.69 (1.26–2.06)	0.831	1.64 (0.89–2.43)	1.75 (1.15–2.33)	0.780
Cortisol	396.9 (275.3–589.8)	497.7 (422.5–646.1)	0.125	401.5 (281.1–620.7)	438.0 (362.2–594.9)	0.688
Cortisone	64.45 (54.37–72.94)	68.96 (60.73–72.81)	0.465	65.86 (52.18–73.92)	63.43 (60.76–68.34)	0.973
**Mineralocorticoids (nM)**						
Corticosterone	10.66 (5.41–23.55)	14.76 (11.62–21.75)	0.144	10.83 (5.90–24.80)	13.61 (9.86–20.21)	0.493
**Tumor biomarkers**						
CA-125 (kU/L)	20.26 (13.10–30.75)	39.66 (21.37–57.32)	**0.018**	21.82 (12.37–34.85)	23.99 (19.69–35.77)	0.285
HE4 (pmol/L)	79.46 (61.84–103.92)	131.51 (114.60–366.27)	**0.001**	79.46 (61.35–114.95)	118.95 (87.42–188.53)	**0.004**

Statistical comparisons are done with Mann–Whitney U test for independent samples. *p* values below the significance threshold 0.05 are bolded. ^a^ Deep myometrial invasion: yes if >50% of myometrium; no if <50% or no MI. 11OHA4, 11β-hydroxy-androstenedione; 11OHT, 11β-hydroxy-testosterone; 11KA4, 11-keto-androstenedione; 11KT, 11-keto-testosterone; A4, androstenedione; CA-125, Cancer Antigen 125; DHEA, dehydroepiandrosterone; HE4, human epididymis protein 4; IQR, interquartile range; LVSI, lympho-vascular space invasion; MI, myometrial invasion: T, testosterone.

## Data Availability

Data will be made available upon reasonable request to the corresponding author.

## Supplementary Materials

The following supporting information can be downloaded at: https://www.mdpi.com/article/10.3390/cancers17101679/s1, Figure S1: Measured steroid hormones (blue boxes) and their positions and relations in steroid biosynthesis; Table S1: Histopathological characteristics of patients with EC included in this study; Table S2: Clinical-pathological characteristics related to median steroid hormone levels (interquartile range) in patients with EC; Table S3: Logistic regression models for EC diagnosis; Table S4: Evaluation of best performing multivariate diagnostic models with bootstraping; Table S5: Logistic regression models for predicting LVSI; Table S6: Logistic regression models for predicting deep MI; Table S7: Logistic regression models for preoperative risk assessment.

## Abbreviations

The following abbreviations are used in this manuscript:

11KA4	11-keto-androstenedione
11OHA4	11β-hydroxy-androstenedione
11OHT	11β-hydroxytestosterone
A4	Androstenedione
AUC	Area under the receiver operating curve
BMI	Body mass index
CA-125	Cancer antigen 125
DHEA	Dehydroepiandrosterone
DHT	5α-dihydrotestosterone
dMMR	missmatch repair deficient
DSS	Disease-specific survival
EC	Endometrial cancer
ESI	Electrospray ionization
FIGO	International Federation of Gynecology and Obstetrics
HE4	Human epidydymis protein 4
IQR	Interquartile range
LC-MS/MS	Liquid chromatography–tandem mass spectrometry
LLOQ	Lower limit of quantification
LVSI	Lymphovascular space invasion
MI	Myometrial invasion
MRI	Magnetic resonance imaging
MTBE	tert-butyl methyl ether
NSMP	Non-specific molecular profile
PCOS	Polycystic ovary syndrome
QC	Quality control
REM	Risk of Endometrial Malignancy
SMAC	Steorid Metabolome Analysiss Core
T	Testosterone
