# Supplementary figures and images for: Multi-Steroid Profiling and Machine Learning Reveal Androgens as Candidate Biomarkers for Endometrial Cancer Diagnosis: A Case-Control Study
Source: Cancers (Basel). 2025 May 16;17(10):1679. doi: 10.3390/cancers17101679 (PMC12110686; doi:10.3390/cancers17101679)

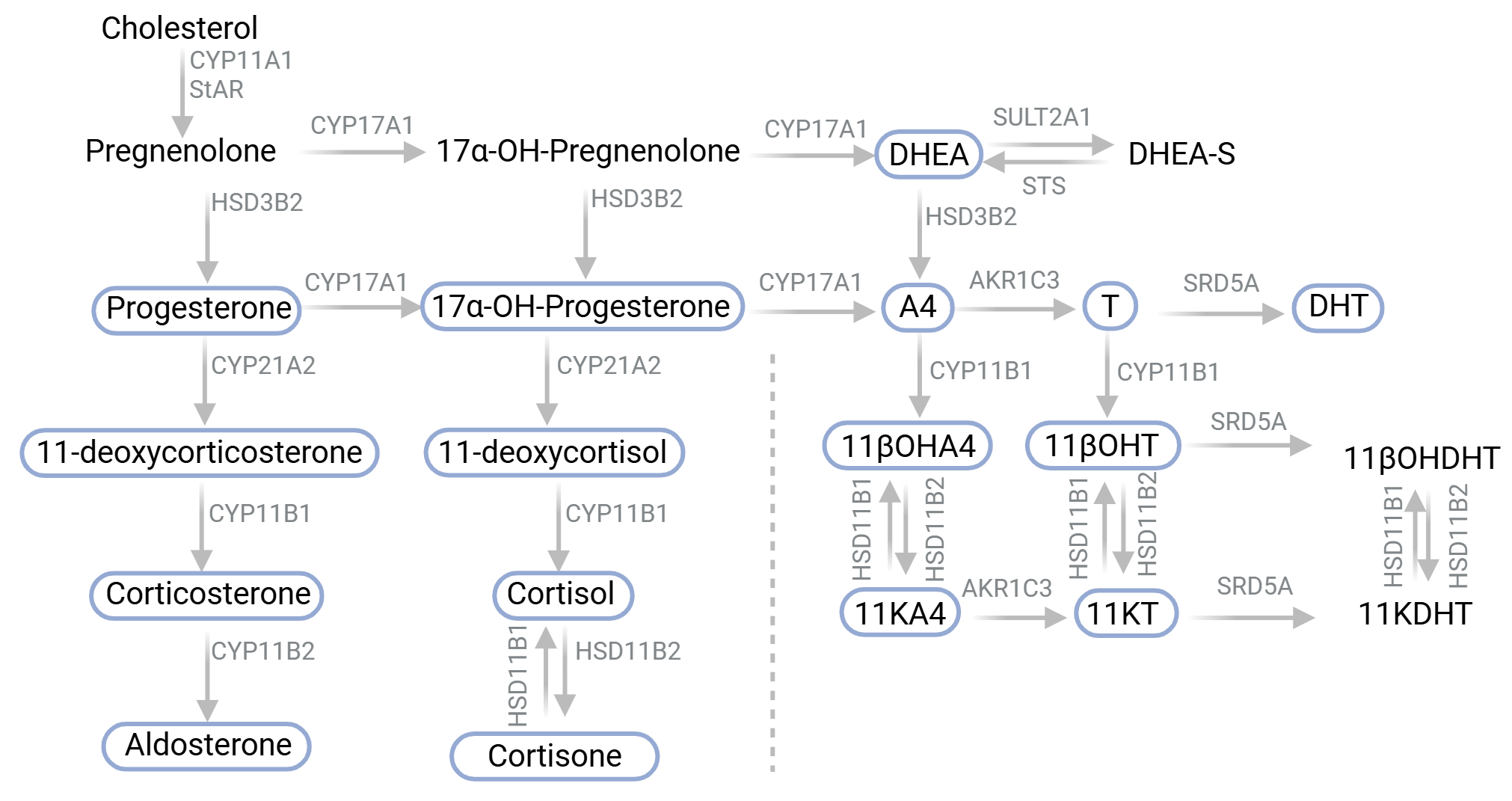

Supplement: Supplementary file 1 [file cancers-17-01679-s001.zip › Supplementary_Figure S1_EC - LCMSMS.png]
